# The past and present of prostate cancer and its treatment and diagnostics: A historical review

**DOI:** 10.1177/20503121231216837

**Published:** 2023-12-01

**Authors:** Miikka Lehtonen, Pirkko-Liisa Kellokumpu-Lehtinen

**Affiliations:** 1 Faculty of Medicine and Health Technology, Tampere University, Tampere, Finland; 2 Research, Development and Innovation Center, Tampere University Hospital, Tampere, Finland

**Keywords:** Prostate cancer, history, diagnostics, treatment

## Abstract

The prognosis of local prostate cancer has improved drastically during the past 60 years. Similarly, the prognosis in metastatic stage is constantly improving due to a number of new pharmaceuticals introduced over the past 10 years. Previously, only palliative treatments were available for prostate cancer, but today, there are multiple options for treatment with curative intent: robotic-assisted radical prostatectomy, stereotactic radiotherapy and brachytherapy. Additionally, life-prolonging chemotherapeutic and androgen-suppressive treatments, as well as diagnostic imaging and staging, have improved considerably. This review summarizes the history of the treatment and diagnostics of prostate cancer, with a focus on the past 60 years. The aim was to provide a concise and easy-to-read introduction on the matter for all people that work with prostate cancer, as well as for patients. The literature was thoroughly examined covering the period from the earliest traceable records to the latest state-of-the-art studies.

## Introduction

The evolution of prostate cancer (PC) treatment has been a success story. In the 1960s, 50%−60% of Northern European men diagnosed for PC died of it within 5 years, and the relative survival rate was still well below 70% in the 1980s.^1,2^ In 2016, the 5-year relative survival rate was 93.6% in Sweden and 92.4% in Finland.^2,3^ In this paper, we will discuss how this remarkable achievement was established by describing key moments in PC discovery and treatment throughout history. The purpose of this review was to provide a concise and easy-to-read introduction on the historical evolution of the treatment and diagnostics of PC. To our knowledge, there has not been a similar review that covers the matter to this depth in over 20 years.^4^ Understanding the progress is both important and interesting not only for urologists, oncologists, radiologists, and pathologists who work with PC, but also for patients with PC and other medical experts, such as nursing staff.

## From ancient times until the 20th century

The first person to describe the prostate was probably the Greek Herophilus of Chalcedon in third century BC, who made his career in Alexandria and whose contributions are known only by indirect references made by Galen.^5,6^ The discovery of the prostate is usually attributed to the Venetian anatomist Niccolò Massala, who described it in his work Anatomiae: liber introductorius from 1536.^5,7^

The earliest biochemically confirmed case of PC occurred in present-day Siberia in the seventh century BC as found in mummified remains of an Iron Age Scythian king exhibiting bone lesions compatible with PC bone metastases.^8^ Biochemical confirmation was performed by detecting positive antibodies against prostate-specific antigen (PSA) and PSA-bound alpha1-antichymotrypsin.^8^ A biochemically unconfirmed case was found as early as 4500 years BC, also in Siberia.^8^

The credit for describing PC has sometimes been given to Londoner surgeon John Adams (1805−1877), who described it as “scirrhous of the prostate gland” in 1853 after a 59-year-old patient had died 3 years after the onset of the disease.^4,9–11^ This was the first case in which cancer was confirmed histologically on autopsy.^9^ However, the German S. Beling described a case of PC leading to mortality in 1822, and the French surgeon Tanche described five cases in 1844.^9^ Adams considered the disease to be very rare at the time.^9,10^

Prussian-born Theodor Billroth performed the first partial prostatectomy in Vienna in 1867.^12^ In 1904, Hugh Hampton Young performed the first radical prostatectomy (RP).^4,12^ In both instances, the surgery was performed through the transperineal approach.^4,12^ Young subsequently reported the results from 19 prostatectomies, with almost complete symptomatic recovery in 15 patients.^13^ One patient lived beyond 5 years after the operation and was presumed to be cured.^13^ Young also performed surgical castration for two patients, but in this case, the results were considered to be negative.^13^

In 1895, Wilhelm Roentgen discovered X-rays.^14^ This was followed by the discovery of naturally occurring radioactivity by Henry Becquerel in the following year through the work he conducted with uranium salts.^15^ In 1898, Marie and Pierre Curie discovered radium and polonium.^16^ The first attempts to cure prostate cancer with radiation were made 10 years later, when the Frenchman Henri Minet published the first results of treating PC with radium (Ra)-containing tubes inserted through urethral or suprapubic catheters in 1909.^4,17^ Therefore, brachytherapy (BT) is actually the oldest form of radiation therapy used to treat prostate cancer. In the next decade, Hugh Hampton Young as well as urologist Octave Pasteau with radium therapist Paul-Marie Degrais published their own results.^4,17^ However, early techniques were difficult to perform and painful for the patient, and thus, internal radiation therapy did not gain interest as a treatment modality for many decades.^4^

**Figure 1.**
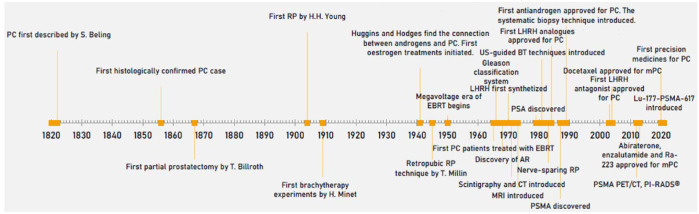
The milestones of the development of PC treatment and diagnostics. AR: androgen receptor; BT: brachytherapy, EBRT: external beam radiotherapy; CT: computer tomography; LHRH: luteinizing hormone releasing hormone; PC: prostate cancer; PET: positron emission tomography; PSA: prostate-specific antigen; PSMA: prostate-specific membrane antigen; RP: radical prostatectomy; US: ultrasound.

The first biomarker found to be useful in PC diagnosis (albeit only in the metastatic stage) was prostate-specific acid phosphatase (PAP), which was discovered by Gutman and Gutman in 1938.^18,19^ The main events of the modern era are illustrated as a demonstrative timeline diagram in Figure 1.

## Huge steps forwards: The era from the 1940s to the 1980s

In 1941, future Nobel laureates from the University of Chicago, Charles Huggins and Clarence V. Hodges, demonstrated that estrogen injections delayed the progression of metastatic cancer.^20,21^ They also showed that testosterone injections accelerate progression.^20^ In the same year, Huggins and Hodges, along with R.E. Stevens Jr, published their first positive results in patients treated with either pharmaceutics (estrogen) or surgical castration.^4,22^

The retropubic approach to prostatectomy was introduced in 1945, when Terrence Millin from All Saints Hospital in London reported the technique.^4,23^ Millin’s technique allowed a more accessible route to the pelvic lymph nodes that could be used for staging.^4^ It remained a mainstay of PC surgery for almost 40 years until 1983, when Walsh et al.^24,25^ developed a nerve-sparing technique for RP.^4^

**Figure 2.**
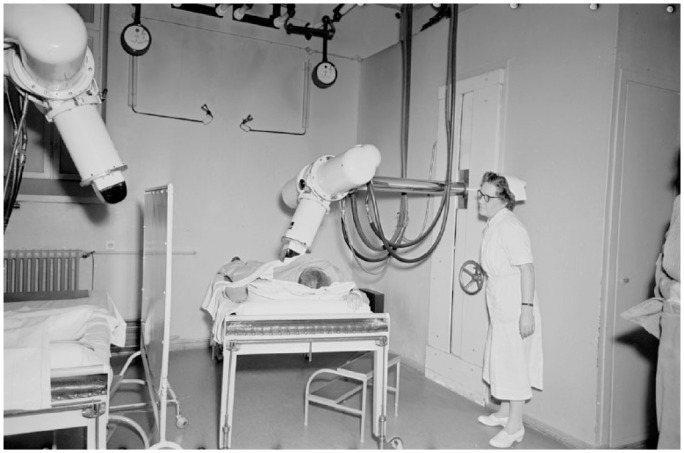
A patient preparing for radiation therapy in Helsinki in 1955, accompanied by a radiation oncology nurse. Photo by Yrjö Lintunen. Published with the permission of Yrjö Lintunen Foundation and Finnish People’s Archives (kansanarkisto.fi).

Until the 1950s, there were no X-ray tubes that could produce radiation capable of penetrating into deeper tissues such as the prostate, and thus, external beam radiotherapy (EBRT) with X-ray machines was mainly used to treat only superficial malignancies and other medical conditions.^4,26^ The period from 1950 onwards is called the megavoltage era of radiation therapy and was characterized by the use of linear particle accelerators and their predecessor, cobalt teletherapy.^26^ In January 1965, George et al.^27^ reported the first patients with inoperable PC to be treated with cobalt therapy.^4^ An example of the contemporary EBRT machinery is shown in Figure 2.

Later, in 1965, Bagshaw et al.^28^ published their results of a trial in which 81 patients with inoperable no distant cancer spread (M0) PC were treated by linear supervised EBRT. The 5-year survival rate was 54%, which was considered excellent at the time.^28^ Bagshaw’s trial was followed by several others, and by the early 1980s, EBRT had become an acceptable treatment modality for PC.^29,30^ The development of intensity-modulated radiation therapy (IMRT) was based on the work by Anders Brahme and others at Karolinska Institute, Stockholm, in the 1980s.^31,32^ It became a mainstay of EBRT in the treatment of PC decades later.^32^

Interest in brachytherapy resumed in the 1970s, when Basil Hilaris and Willet Whitmore Jr, working for Memorial Sloan Kettering Cancer Center, reported a new technique utilizing iodine-125 isotopes.^4,17^ Their technique did not require any image guidance.^4,17^ Although the method was initially popular, it was later discarded due to the high rate of long-term failure and complications.^4,17^ In the 1980s, Hans Henrik Holm from Denmark developed a technique in which brachytherapy seeds were implanted through transrectal ultrasound (TRUS) guidance, which finally led to a breakthrough in the technique and its adaptation to clinical practice.^4,17^

### Androgen deprivation from the 1970s to the 1980s

The structure of luteinizing hormone releasing hormone (LHRH) and the methods for synthesizing it were discovered by a research group led by Andrew V. Schally from Tulane University School of Medicine and published in 1970 and 1971.^33^ For this discovery, Schally was awarded a Nobel prize in 1977.^4^ Sandow et al.^34^ demonstrated that treatment with an LHRH analog suppressed testosterone production in rats after an initial surge in 1978. A research group including Schally, among others, demonstrated the beneficial effect of LHRH analog treatment in patients with prostate cancer in 1982.^35^ The first LHRH analogs approved for commercial use in PC were buserelin and leuprolide in 1984.^36^ LHRH analogs remain one of the most commonly used alternatives for androgen deprivation therapy (ADT) in the treatment of PC to date.

After the discovery of the androgen receptor in 1968, this receptor was also a tempting target for drug developers.^37^ However, the first antiandrogen, cyproterone, proved to be unsuccessful since it crossed the blood-brain barrier and blocked the androgen receptors of the brain (leading to increased secretion of LH) in addition to blocking the receptors in the testicles.^4^ This issue was overcome by adding an acetate group to the molecule, thus creating cyproterone acetate, which was approved by the Food and Drug Administration (FDA) for the treatment of PC in 1989.^4^

Although the first chemotherapy agents for cancer, aminopterin and nitrogen mustard, were introduced in the 1940s,^38^ PC remained an obstacle for chemotherapeutics for a long time. The first chemotherapeutic agent that was found to be useful for PC was estramustine in 1981.^39^ Estramustine acts as a microtubule-stabilizing agent but also has estrogenic properties and is in fact a derivative of estradiol formed through an addition reaction with nor nitrogen mustard.^39–41^ Although estramustine improved biochemical recurrence-free survival (BRFS), it was not shown to be clearly beneficial in regard to overall survival.^39^ In addition, troublesome side effects, such as nausea and cardiovascular toxicity, also limit its use.^42^

### Advancements in diagnostics from the 1960s to the 1980s

In diagnostics, this 40-year era is especially remembered for the Gleason histopathological grading system published by Donal F. Gleason in 1966.^43–45^ It gradually replaced the preceding Broders classification system from 1926.^46^ PSA was discovered in 1979 by a research team led by Ming Chang Wang from Roswell Park Cancer Institute in Buffalo, New York.^47,48^ Eight years later, Stamey et al.^49^ demonstrated its usefulness as a biomarker in PC and benign prostate hyperplasia.^48^ PSA’s sensitivity greatly exceeded that of PAP and was also found to be useful in local staging diagnostics.^49^ PSA testing as a method of evaluating treatment response became a clinical practice in the United States in the 1980s and became used as a diagnostic tool in the following decade.^50^

The systematic biopsy technique was introduced in 1989 by Hodge et al.^51,52^ In this technique, the urologist biopsies particular anatomical sites systematically under TRUS guidance, even if there were no lump or abnormal firmness palpable.^51^ The systematic technique improved the sensitiveness of detecting PC and replaced the previous techniques which relied on urologist’s palpation findings and ultrasound interpretation.^51,52^ The systematic technique still recommended additional targeted biopsies of the suspicious areas.^51^ The original technique included six cores.^51,52^ The 12-core system became a standard approximately 15 years later.^52^

In the field of imaging, the principles of using radioisotopes to detect metastases were also introduced in the 1960s.^53,54^ The technique of producing bone scintigraphy images by detecting metastable technetium-99 isotopes with gamma cameras was introduced by Subramanian and McAfee in 1971 and is still in use today.^53,54^ In the same year, the first patients were imaged with computer tomography invented by Sir Godfrey Hounsfield and Alan M. Cormack, Nobel laureates of 1979.^55^ MRI imaging was introduced in 1973 by Paul Lauterbur, Nobel laureate of 2003.^55^

## The past 30 years: The revolution of chemotherapy, nuclear medicine and more

In 1996, a new chemotherapeutic, mitoxantrone, was introduced to treat metastatic castration-resistant PC (mCRPC).^56^ However, mitoxantrone was shown to improve only palliative endpoints and not overall survival (OS).^56^ The groundbreaking year was 2004, when the SWOG 99-16 and TAX-327 trials showed that docetaxel improved OS either in combination with estramustine or alone.^57,58^ According to current knowledge, the added value of estramustine seems to be low compared with its increased toxicity,^59^ and it is rarely used. In 2015, the E3805 study (which is often also referred to as the “CHAARTED” trial) showed that docetaxel was also beneficial for metastatic hormone-sensitive prostate cancer (mHSPC).^60,61^ The only other chemotherapeutic that has been shown to improve OS in metastatic PC is cabazitaxel, which is a taxane chemically similar to docetaxel.^62^ It was shown to improve survival in mCRPC as a second-line treatment after docetaxel failure compared with palliative mitoxantrone in the TROPIC trial in 2010.^63^ Both taxanes exert their cytotoxic effect on cancer cells by stabilizing microtubules.^64^

In ADT, the first LHRH antagonist to be approved was abarelix in 2003.^65^ Two years later, the drug was withdrawn due to concern over hypersensitivity reactions.^65^ However, other LHRH antagonists, such as degarelix and relugolix, remain on the market.^66^ They have the benefit of avoiding the “flare” reaction associated with LHRH agonists, although other benefits remain unclear, and no long-term suspensions are available, meaning at least monthly injections are needed.^66^

Unlike LHRH antagonists, a novel group referred to as androgen receptor pathway inhibitors (ARPIs) was introduced in the 2000s and has been shown to improve survival compared to conventional treatments in mCRPC, mHSPC and nonmetastatic castration-resistant PC (nmCRPC).^66^ ARPIs include abiraterone acetate, enzalutamide, apalutamide, and darolutamide.^66^ Enzalutamide, apalutamide and darolutamide resemble first-generation antiandrogens in their mechanism of action but bind with greater affinity to ARs and hinder the receptor’s translocation into the nucleus, unlike first-generation antiandrogens.^67^ Abiraterone acetate is different and mainly affects the production of extragonadal androgens.^68^ Abiraterone and enzalutamide were the first to be approved (in mCRPC) in 2013.^39^ The key drug trials of the present millennium are summarized in Table 1. Recently, darolutamide became the first ARPI to demonstrate a survival benefit in combination treatment with docetaxel in mHSPC with the results from the ARASENS trial from 2022.^69^

**Table 1.** The key drug trials of the 21st century summarized.

Trial name	Stage	Intervention versus control	End-result	Year	Refs
SWOG 99-16	mCRPC	Docetaxel + estramustine versus mitoxantrone	Docetaxel becomes approved for mCRPC	2004	55
TAX-327	mCRPC	Docetaxel versus mitoxantrone	Docetaxel becomes approved for mCRPC	2004	56
IMPACT	mCRPC	Sipuleucel-T versus placebo*	Sipuleucel-T shown useful in mCRPC	2010	112
COU-AA-301	mCRPC	Abiraterone versus placebo**	Abiraterone approved for mCRPC after treatment failure with docetaxel	2012	113
AFFIRM	mCRPC	Enzalutamide versus placebo**	Enzalutamide approved for mCRPC after treatment failure with docetaxel	2012	114
ALSYMPCA	mCRPC	Radium-223 dichloride versus placebo**	Radium-223 dichloride approved for mCRPC after treatment failure with docetaxel	2013	72
TROPIC	mCRPC	Cabazitaxel versus mitoxantrone**	Cabazitaxel approved for mCRPC after treatment failure with docetaxel	2013	115
COU-AA-302	mCRPC	Abiraterone versus placebo*	Abiraterone approved for docetaxel-naïve mCRPC patients	2015	116
E3805 (‘CHAARTED’)	mHSPC	Docetaxel + ADT versus ADT	Docetaxel shown useful in high burden mHSPC	2018	117
LATITUDE	mHSPC	Abiraterone versus placebo	Abiraterone becomes approved for mHSPC	2019	118
PROfound	mCRPC	Olaparib versus enzalutamide/abiraterone*	Olaparib approved for patients with *BRCA* or *ATM* mutations	2020	119
PREVAIL	mCRPC	Enzalutamide versus placebo*	Enzalutamide approved for docetaxel-naïve mCRPC patients	2020	120
PROSPER	nmCRPC	Enzalutamide versus placebo	Enzalutamide becomes approved for nmCRPC	2020	121
ARAMIS	nmCRPC	Darolutamide versus placebo	Darolutamide becomes approved for nmCRPC	2020	122
VISION	mCRPC	Lutenium-177-PSMA-617 versus standard of care**	Lutenium-177-PSMA-617 approved for mCRPC	2021	71
SPARTAN	nmCRPC	Apalutamide versus placebo	Apalutamide becomes approved for nmCRPC	2021	123
TITAN	mHSPC	Apalutamide versus placebo	Apalutamide becomes approved for mHSPC	2021	124
ARASENS	mHSPC	Darolutamide + docetaxel versus docetaxel	Darolutamide shown useful in combination treatment of mHSPC	2022	67
ARCHES	mHSPC	Enzalutamide versus placebo	Enzalutamide shown to increase survival in mHSPC	2022	125

mCRPC: metastatic castration-resistant PC; mHSPC: metastatic hormone-sensitive PC; nmCRPC: nonmetastatic castration-resistant PC; PSMA: prostate-specific membrane antigen.

* Prior docetaxel, **after treatment failure with docetaxel.

### The era of prostate-specific membrane antigen and nuclear medicine

In 1987, Horoszewicz et al.^70^ from the State University of New York identified a novel antigen in the LNCaP cell line. In 1994, this antigen was named prostate-specific membrane antigen (PSMA) by Israeli et al.^71^ Thirty years later, radiolabelled PSMA molecules are becoming a standard in both PC imaging and anticancer therapy of mCRPC.^72,73^ In diagnostic imaging, PSMA PET/CT scans have been performed since approximately 2012 and have been determined to improve both sensitivity and specificity in staging when compared with MRI, standard PET, scintigraphy or CT.^72^ The use of the modality continues to increase.

In 2013, the ALSYMPCA trial first showed that radioactive isotopes could also be used to treat mCRPC.^74^ The trial used radium-223.^74^ The VISION trial in 2021 used a radioactively labelled PSMA molecule (lutetium-177-PSMA-617) that further increased survival.^73^

### Developments in precision medicine and immuno-oncology

Although the present era in general oncology has been a triumph for precision medicine drugs,^75^ developments in the treatment of PC have remained modest at best.^76^ However, some steps forward have been taken. Currently, the European Association of Urology (EAU), National Comprehensive Cancer Network (NCCN^®^) and American Urology Association (AUA) recommend genetic testing when possible, at least in metastatic PC.^66,77,78^ The poly ADP-ribose polymerase (PARP) inhibitor olaparib was shown to increase the survival of *BRCA1/BRCA2/ATM* mutation carriers with mCRPC in the PROfound trial in 2020.^79^

In immuno-oncology, success has been even more limited. FDA approved sipuleucel-T in the treatment of minimally symptomatic mCRPC patients in 2010.^80^ This “cancer vaccine” manufactured from the patient’s own cancer cells is not available in Europe.^66,80^ The usual immuno-oncologic approaches based on industrially manufactured cancer antibodies have not been proven to improve survival thus far. A phase II study showed promising results with pembrolizumab,^81^ but the first phase III study was discontinued due to negative intermediate results.^82^

### The past few decades of curative treatments and active surveillance

In both PC surgery and radiotherapy, advancing technology has played a major role. Laparoscopic prostatectomy techniques were developed in the early 1990s as an alternative to Walsh’s technique.^83^ Robotic-assisted prostatectomy (RAP) was introduced in approximately 2001,^83,84^ and while Walsh’s technique remains equal in OS and other primary endpoints,^66^ RAP seems to reduce operative bleeding and increase surgeon comfort.^84,85^ Other developments in the RP field have been the introduction of salvage radiotherapy in patients with biochemical failure,^86^ as well as adjuvant radiotherapy for those with negative features after surgery, such as positive margins or extracapsular extension.^87^

In EBRT, advances in radiotherapy machinery have enabled more accurate dose planning. First, image guidance systems have become available, meaning more accurate patient positioning at every treatment visit.^88^ Second, the treatment areas can now be designed with more asymmetrical borders thanks to intensity-modulated radiotherapy (IMRT) or volumetric arc therapy (VMAT) techniques, sparing healthy tissues.^89,90^

The developments in EBRT technology leading to more accurate dose planning have also encouraged the investigation of higher doses per fraction.^91^ In the 2010s, moderate hypofractionation with a 2.5−3.4 Gy fraction size was investigated in four trials,^92–94^ three of which reported noninferior toxicity and survival results.^92–94^ In 2019, a Cochrane meta-analysis concluded that moderate hypofractionation is indeed noninferior.^95^ Since moderately hypofractionated therapy is more cost effective with equal outcomes,^96^ it is currently the gold standard of EBRT recommended by the EAU.^66^

The reporting of quality of life (QoL) results started to become mainstream in the 1990s.^97^ Since both RP and EBRT for PC decrease the patient’s QoL at least in some ways,^98^ the question of how to prevent or delay the negative impacts on patient QoL was raised, as low-risk cancers are unlikely to affect the patient’s OS.^99^ As a response, the concept of active surveillance of local PC was developed to defer possibly needless active treatment, supported by the randomized trial ProtecT, which showed that deferring treatment until it was deemed necessary did not decrease survival in low- or intermediate-risk PCs.^100^ The concept of active surveillance or monitoring has since been integrated into both European and American guidelines.^66,77^

### Further developments in diagnostics

A groundbreaking year in the pathological grading of PC was 2005, when the International Society of Urological Pathology (ISUP) decided that Gleason scores below 5 should not be used.^101^ Although the 2005 conference did not directly comment against the use of the Gleason 5 score,^101^ its use gradually declined. For example, in Sweden, the use of the Gleason 5 score declined from 7.4% of cancerous biopsy samples to 0.9% in 2011.^102^

Since Gleason scores were now only reported from 6 or more and patterns 1−2 were not used, the 2014 ISUP conference proposed a new classification system, which reclassified Gleason scores 6−10 into corresponding ISUP grade groups 1−5.^103^

A major development in PC diagnostics in the 2010s was the performance of MRI prior to biopsy to reduce the number of needless biopsies.^66^ The European Society of Urogenital Radiology (ESUR) released a version of the PI-RADS^®^ classification system for prostate MRI lesions in 2012.^104^ Since the 2010s the techniques that incorporate MRI findings, TRUS and palpation findings (so called cognitive fusion biopsies), as well as directly MRI-guided biopsies have become a golden standard in PC diagnostics,^66,105^ even though the systematic biopsies are still recommended in addition except for selected patients with prior negative biopsies.^66^

## Discussion: The future

Overall, the treatment of PC has taken huge leaps forwards. In Finland, the reported cancer-specific survival of local cases in 2020 was approximately 98% 5 years after diagnosis.^106^ With these results, it may be best for future research to focus the limited resources on finding solutions to improve the prognosis of high-risk and metastatic cases. As the past 10 years have proven, the treatment of metastatic PC is constantly improving and providing promising results for future patients.

In EBRT, stereotactic body radiation (SBRT) has been a topic of interest over the past 10 years.^107,108^ This ultra hypofractionated form of radiotherapy with a fraction size ⩾3.5 Gy has not been inferior in terms of survival thus far,^66,107^ although long-term data are still needed. However, the present data show it to have inferior QoL in the short-term compared with conventional radiation,^107^ which raises questions as to whether it could replace moderately hypofractionated radiation.

In brachytherapy, high-dose rate BT (HDR BT) with temporarily implanted catheters has been investigated in recent decades as an adjuvant therapy with EBRT in a study conducted in Mount Vernon Hospital, UK.^109^ While HDR BT boost improved BRFS, it did not improve OS even in the final results after 12 years.^109^ Prostate cancer-specific survival data were not collected.^109^ There was no difference in toxicity.^109^ If BT is to develop any further, these types of adjuvant treatments reducing the number of hospital visits required for EBRT even further would be one option. BT boosts could still be useful in selected patients, such as those who are younger than an average PC patient, have few comorbidities and would benefit from improved BRFS, which would reflect survival perhaps only after 15–20 years. This remains a question for future studies.

In surgery, the usefulness of prostatectomy for the primary tumor in oligometastatic disease has been debated, and retrospective studies have shown promising results.^110^ The results for EBRT have been promising in the STAMPEDE and HORRAD trials, with a benefit in newly diagnosed patients with a low-tumor burden in the STAMPEDE trial,^111,112^ but a randomized trial has not yet investigated RP.^113^ Future trials have been encouraged by the scientific community.^113^ Also, minimally invasive and well-tolerated procedures such as photodynamic therapy may challenge active surveillance if proven effective in future trials.^114^

In the pharmaceutical management of PC, the large majority industry-financed phase II and phase III trials now focus on immunotherapy, precision medicine and theranostics (drugs that combine imaging and diagnostics similar to Lu-177-PSMA-617).^115^ One trial whose final results are waited in the near future, is the TRITON3 investigating PARP-inhibitor rucaparib in metastatic PC.^116^ Recently it was shown to improve progression-free survival against the drug of physician’s choice.^116^ In the imaging, the desire to find new radiotracers, as well as further investigate PSMA PET/CT are likely to remain as a matter of interest. One promising tracer is a bombesin antagonist gallium-68-RM2 which may provide added value in detecting bladder and pancreatic metastases.^117^ In MRI, radiomics and deep learning models are developed to aid in radiologist’s work.^118^

PSA screening was a topic of interest between the 1990s and the 2010s. The European Randomized Study of Screening for PC (ERSPC) included eight countries and 182,000 participants who were 55–69 years old.^119^ There have also been other randomized trials of considerable size.^120^ Despite tremendous effort, PSA screening has been shown to be ineffective in reducing overall mortality and seems to have only a minor effect on cancer-specific survival.^120^ The focus in screening studies has now shifted to MRI-based screening studies, and large trials using this approach are now being conducted, such as GÖTEBORG-2 in Sweden.^121^

## Limitations

This article is a historical review that aimed to provide a broad introduction on the history of treatment and diagnostics of PC instead of analyzing any particular question in-depth. The authors come from medical background, and no professional historian took part in the writing of the article.

## Conclusions

The evolution of local PC treatment has been a triumph of modern medicine, which showed that the prognosis of usually incurable, fatal disease can be overturned in only approximately 60 years. The focus of research has now shifted towards avoiding and deferring the harms of treatment. Metastatic PC remains a challenge, but also its prognosis has steadily improved, and new treatments are introduced at regular intervals. The advancements in the diagnostics, such as PSMA PET/CT and PI-RADS^®^ classification, aid in the fight against PC and timely treatment choices.
